# Our Journal

**Published:** 1848-02

**Authors:** 


### PART V.—EDITORIAL.

### ARTICLE I.

### OUR JOURNAL.

Our Journal, as will be seen by the prospectus accompanying this the last number of the volume, is to be continued hereafter, as the North Western Medical and Surgical Journal, a title suggested by a number of our friends and patrons, as the most appropriate one for a medical periodical intended for and sent already into all parts of the North West to more than six hundred subscribers.

In view of the liberal patronage already extended to us, we are led to anticipate a large addition to our subscription list during the coming year, and in order to meet the demand we shall print at least 1000 copies of the forthcoming volume, at an additional expense, which can only be met by an extended circulation and prompt payment, especially from those still in arrears for the volumes already published.

We shall endeavor on our part, by renewed efforts in furnishing matter and making selections, such as will be most useful and interesting to north-western medical men, to sustain the present character of our Journal for being peculiarly well adapted to the wants of practitioners in this region.

All we ask is a continuation of the disposition now manifested by the profession, to aid us in furnishing interesting matter for publication, and the payment voluntary of the small amount required annually to "pay the printer."

Subscribers who are still indebted for the volume just completed, or the preceding one, will receive their bills with this number; and unless prompt payment is made, we shall be compelled in order to pay expenses, to place them at once in other hands for collection.

Payments, made by mail at our risk, will be acknowledged in the list of receipts, which will be published hereafter, from time to time, upon the cover of the Journal.

